# Accurate prediction of RNA 5-hydroxymethylcytosine modification by utilizing novel position-specific gapped k-mer descriptors

**DOI:** 10.1016/j.csbj.2020.10.032

**Published:** 2020-11-12

**Authors:** Sajid Ahmed, Zahid Hossain, Mahtab Uddin, Ghazaleh Taherzadeh, Alok Sharma, Swakkhar Shatabda, Abdollah Dehzangi

**Affiliations:** a Department of Computer Science and Engineering, United International University, Dhaka, Bangladesh; b Department of Natural Science, United International University, Dhaka, Bangladesh; c Institute for Bioscience and Biotechnology Research, University of Maryland, College Park, MD 20742, USA; d Institute for Integrated and Intelligent Systems, Griffith University, Brisbane, QLD 4111, Australia; e Department of Medical Science Mathematics, Tokyo Medical and Dental University (TMDU), Tokyo, Japan; f Laboratory for Medical Science Mathematics, RIKEN Center for Integrative Medical Sciences, Yokohama, Japan; g School of Engineering and Physics, University of the South Pacific, Suva, Fiji; h Department of Computer Science, Rutgers University, Camden, NJ 08102, USA; i Center for Computational and Integrative Biology, Rutgers University, Camden, NJ 08102, USA

**Keywords:** RNA 5-hydroxymethylcytosine modification, Sequence-based feature, Position-specific gapped k-mer, Position-specific k-mer, Logistic regression

## Abstract

RNA modification is an essential step towards generation of new RNA structures. Such modification is potentially able to modify RNA function or its stability. Among different modifications, 5-Hydroxymethylcytosine (5hmC) modification of RNA exhibit significant potential for a series of biological processes. Understanding the distribution of 5hmC in RNA is essential to determine its biological functionality. Although conventional sequencing techniques allow broad identification of 5hmC, they are both time-consuming and resource-intensive. In this study, we propose a new computational tool called iRNA5hmC-PS to tackle this problem. To build iRNA5hmC-PS we extract a set of novel sequence-based features called Position-Specific Gapped k-mer (PSG k-mer) to obtain maximum sequential information. Our feature analysis shows that our proposed PSG k-mer features contain vital information for the identification of 5hmC sites. We also use a group-wise feature importance calculation strategy to select a small subset of features containing maximum discriminative information. Our experimental results demonstrate that iRNA5hmC-PS is able to enhance the prediction performance, dramatically. iRNA5hmC-PS achieves 78.3% prediction performance, which is 12.8% better than those reported in the previous studies. iRNA5hmC-PS is publicly available as an online tool at http://103.109.52.8:81/iRNA5hmC-PS. Its benchmark dataset, source codes, and documentation are available at https://github.com/zahid6454/iRNA5hmC-PS.

## Graphical abstract

**Figure:**
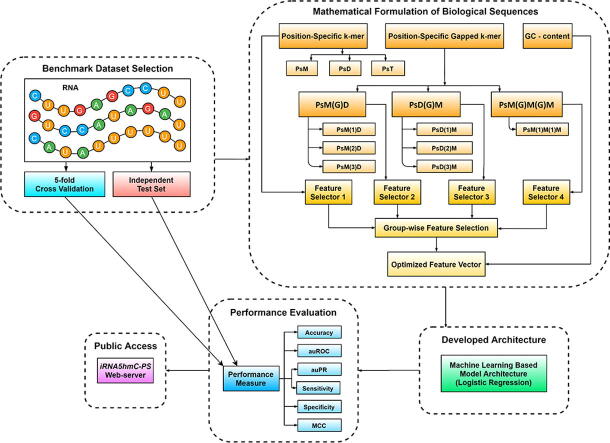

## 1 Introduction

Over the last few decades, a wide variety of RNA-related challenging research problems have been surfaced. Among them, RNA modification is one of the most important and challenging research problems. Since the initial detection of structurally modified nucleoside and pseudouridine in 1950 [1], more than 160 distinct RNA modifications have been identified in mRNA, tRNA, rRNA, and snRNA [2], [3]. These modifications can affect several biological processes, such as transcription, pre-RNA splicing, RNA export, RNA degradation, and mRNA translation [4], [5], [6], [7]. RNA modification has also been identified in association with human diseases such as cancer, cardiovascular diseases (heart attack, stroke, vascular disease, etc.), and diabetes [4], [5]. Therefore, it is of paramount importance to determine their distribution in transcriptomes to assess the biological functionalities of RNA modifications.

Over the past few years, due to the impressive efficiency of high-throughput sequence-based methods, a wide range of studies have been proposed to identify different types of RNA modifications. The most notable modifications include:(1) N^6^-methyladenosine (m^6^A), which is an internal mRNA modification related to the regulation of cellular processes [8], (2) Pseudouridine (Ψ), which is known for influencing pre-mRNA splicing and mRNA translation and/or mRNA stability [9], (3) 5-methylcytidine (m^5^C), that regulates mRNA stability [10], (4) N^4^-acetylcytidine (ac^4^C), that is involved with mRNA translation and stability [11], and (5) N^7^-methylguanosine (m^7^G), which plays a role in transcription elongation, mRNA stability and degradation [12].

Recently, Fu *et al.* used TET-mediated oxidation of m5C to develop a new RNA modification called 5‑Hydroxymethylcytosine (5hmC) [13]. Though 5hmC was first discovered in wheat seedlings [14], later on, it was also discovered in human and mouse tissues such as the brain, heart, and pancreas [15]. After that, both Delatte et al. and Miao et al. experimented on Drosophila and mouse, respectively, and both concluded that 5hmC modifications are enriched in brain tissue [16], [17]. Hence, suggesting that 5hmC modifications are intertwined with brain tissue. Delatte et al. [16] analyzed hydroxymethylation of cytosines in RNA (hmC) in Drosophila melanogaster S2 cells and observed a positive correlation between transcript abundance and hmC peaks. They found a positive correlation between hmC abundance and active mRNA translation by analyzing the distribution of this modification as a function of mRNA translation status. Additionally, in vitro translation analysis of unmodified, methylated, and hydroxymethylated Firefly Luciferase-encoding RNA templates in rabbit reticulocyte lysate suggested that hydroxymethylation can restore translation efficiency of previously methylated substrates. These experimental results suggest that RNA hydroxymethylation has a significant impact on mRNA translation, which was an unknown functionality until that point. Understanding other functionalities of this modification can also provide significant insights related to the field of epitranscriptomics. However, there are still knowledge gaps in understanding the biological functionalities of 5hmC due to the lack of information on its distribution in various species. Although the experimental methods are effective and highly accurate, they are expensive and time-consuming. In order to speed up the process and reduce the cost, it is imperative to develop computational methods for identifying RNA 5hmC sites with equally high accuracy and efficiency.

Most recently, iRNA5hmC was proposed in [18] as the first machine learning-based model to predict RNA 5hmC modifications solely based on RNA sequential information. To build this model, Liu et al. [18] only relied on k-mer frequencies (2-mer and 3-mer) and a position-dependent one-hot vector-based encoding mechanism (each residue in a sequence is substituted with a nucleotide-specific one-hot vector of length 4) for feature extraction purpose. Although class-discriminatory information retention capability of these feature vectors has been demonstrated through rigorous experiments, we started with the hypothesis that exploring more sequence-based feature extraction approaches would increase the possibility of retrieving crucial information, relevant for better class-separation, thus improving the predictive capability of the subsequent classification models.

In this study, we introduce *iRNA5hmC-PS*, a machine learning-based prediction framework that incorporates two types of features namely, (1) “Position-Specific Gapped k-mer” (novel descriptors for RNA sequences presented in this study) and (2) “Position-Specific k-mer” [19], to maximize the retention of class-distinguishing information from RNA sequences. Like *iRNA5hmC*, our features are extracted from RNA sequence information only. Our proposed “Position-Specific Gapped k-mer” features retain position-specific information of both short-range and long-range patterns from RNA sequences. To the best of our knowledge, none of the existing hand-engineered feature extraction techniques for nucleotide sequences provide such capabilities from primary sequence information only.

**Fig. 1:**
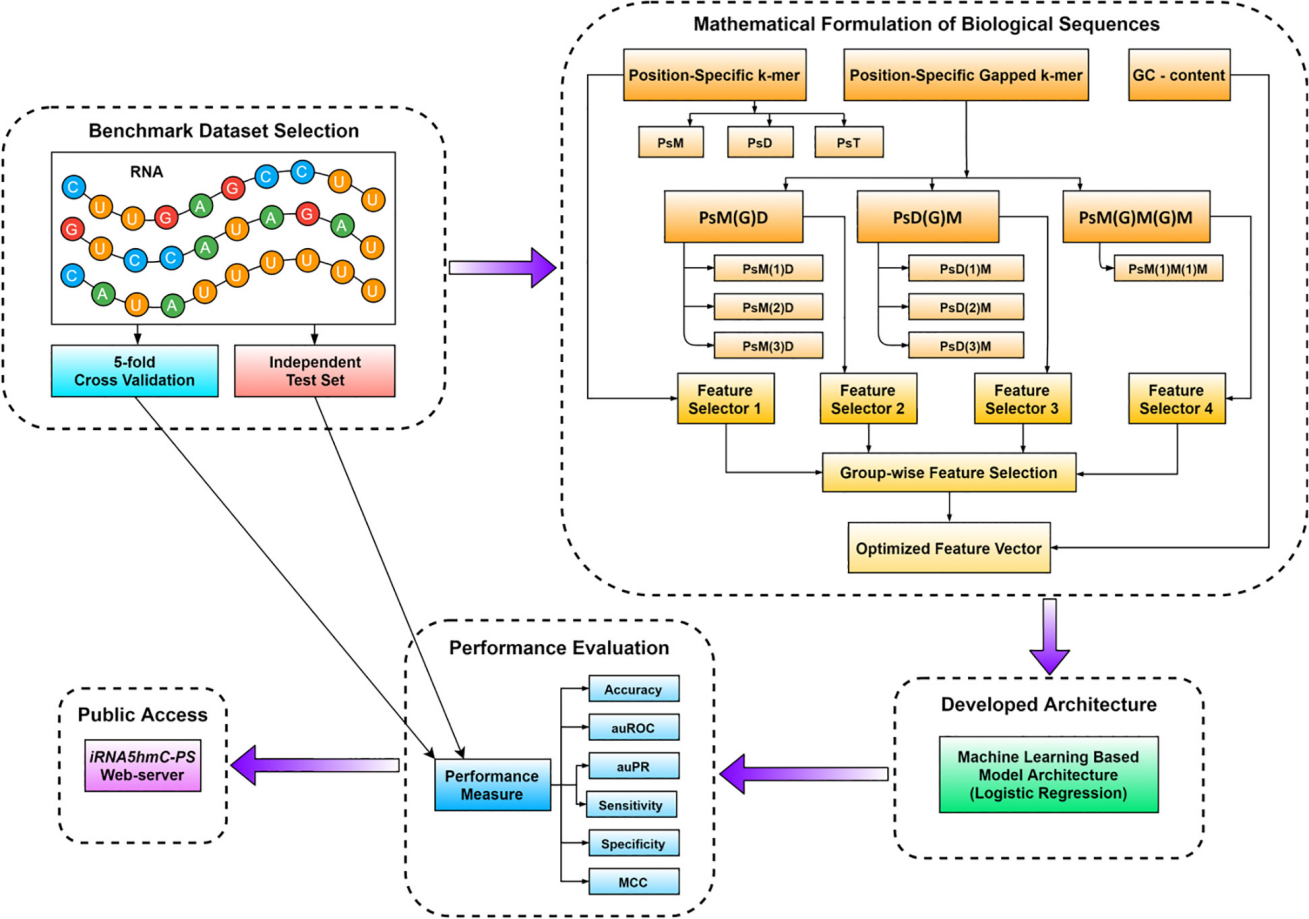
System diagram of *iRNA5hmC-PS.* As shown in this figure, we first select our training and test sets. We then extract our proposed features, train our model, evaluate the model’s generalization capability, and finally deploy the trained model for identifying RNA 5hmC sites. Note that “Feature Selector 1”, “Feature Selector 2”, “Feature Selector 3”, and “Feature Selector 4” refer to four independent Random Forest classification models that select most discriminative features from four different feature groups (Position-Specific k-mer, PsM(G)D, PsD(G)M, and PsM(G)M(G)M), respectively.

Next, *iRNA5hmC-PS* employs a group-wise feature selection component to mitigate the curse of dimensionality by discarding the less informative features. Lastly, it employs Logistic Regression for the classification purpose. Fig. 1 demonstrates the general architecture of *iRNA5hmC-PS* that provides a broad overview of these steps before we delve into their details in the following sections. Experimental results on a publicly available benchmark dataset show that iRNA5hmC-PS is able to outperform the existing state-of-the-art method *iRNA5hmC* with a large margin. *iRNA5hmC-PS* achieves 78.3%, 0.86, 0.86, 80.0%, 79.5%, and 0.56 in terms of Accuracy, auROC, auPR, Sensitivity, Specificity, and MCC, respectively that in all cases are significantly better than those reported results found in the literature. As a result, we believe it has the potential to be an accurate and efficient tool to identify 5hmC sites. *iRNA5hmC-PS* is publicly available as a web-server at http://103.109.52.8:81/iRNA5hmC-PS and benchmark dataset, source codes, and documentation for all the models are available at https://github.com/zahid6454/iRNA5hmC-PS.

## 2 Benchmark dataset

The selection of a standard and reliable benchmark dataset is the key to establishing a high caliber bioinformatics tool [20]. In this study, we have employed the benchmark dataset *D* used in [18] to develop the predictor and evaluate its predictive performance. The dataset contains 1324 samples where 662 are positive and 662 are negative. It can be formulated as,

(1) $$D=D^{+}\cup D^{-}$$

where, D^+^ indicates a set of 41 nt (nucleotides) RNA sequences with an experimentally detected 5 Hydroxymethylcytosine (5hmC) at the central (21st) position and D^−^ indicates a set of 41 nt RNA sequences with a central cytosine that is not detected as 5hmC site. In addition, the operator ∪ indicates the union between the positive and negative sets. Although the number of nucleotides considered on both sides of the central cytosine can be tuned as a parameter based on some objective function, in this study, we have only considered 40 nucleotides since the dataset available in [18] has been preprocessed beforehand based on experiments for RNA m7G site detection task reported in [21]. Although a large number of sequences containing central non-5hmC sites were collected initially, 662 of these sequences were randomly selected for balancing the number of positive and negative samples as it is explained in [18]. To evaluate the model’s generalization capability and to avoid the possibility of performance overestimation, we randomly separated 10% of the samples with label stratification from the original dataset and generated an independent set. The remaining 90% of the samples are used as training and validation sets. After separation, the training and validation sets contain 1192 instances in total, and the independent set contains 132 instances. It is important to note that the independent test set was carefully kept separate as unseen data and was not used in learning and parameter tuning processes.

## 3 Mathematical formulation of RNA sequences

As described in the introduction section, the second step is the formulation of mathematical expressions from biological (RNA sequences for our case) sequences, also known as feature extraction, which is crucial to establish a robust bioinformatics tool [20]. Over the years, a wide range of techniques has been proposed for embedding nucleotide sequences into multi-dimensional feature spaces [22]. However, it remains extremely challenging to come up with a representation scheme with significant discriminatory information for accurate sequence-to-category mapping. This challenge can be attributed to the fact that distinctive local sequence-patterns and their long-range interactions are hard to retain in numerical vector representations. Therefore, to obtain vector representation for the optimal detection of RNA 5hmC sites, in this study, we used two techniques, namely, (1) Position-Specific Gapped k-mer, and (2) Position-Specific k-mer. The following subsections describe these techniques in detail.

### 3.1 Position-specific gapped k-mer

k-mer frequency is one of the most commonly employed features for representing biological sequences [19], [23], [24], [25], [26], [27] including RNA modifications such as identification of Pseudouridine sites [28], [29], m5C RNA sites [30], [31], *N^7^*-methylguanosine sites [21], and 5hmC sites [18]. However, k-mer frequency-based features have limitations. As the value of *k* becomes large for capturing long-range residue interactions, the length of the resulting feature vector increases exponentially, leading to a sparse representation that causes the underlying prediction models to lose generalization capability. To address this issue, Ghandi et al., proposed gapped k-mer frequency-based features which combine similar (this definition of similarity varies across different feature generation modes) sequence-patterns into a single consolidated pattern, thus mitigating sparsity [32]. Ever since then, these gapped k-mer frequencies have been widely used in a series of biological sequence annotation problems [33], [34], [35] including RNA modifications such as identification of N^6^-Methyladenosine sites [36].

Although long-range interaction information preservation capability of the resulting feature vector improves drastically after incorporating frequencies of the gapped patterns, these solely frequency-based features do not encode positional information of the underlying patterns along the sequence. Therefore, the downstream classification models cannot infer the relative position of different patterns from these frequency features. Suppose the positional information of these patterns is retained during the feature extraction process. In that case, the downstream classification functions can exploit their spatial relationship for detecting various contiguous or gapped sequence patterns of any arbitrary length (hypothetically, patterns as long as the whole sequence can be captured by classifiers using the positional information of smaller patterns) that provide further class-discriminatory information. For biological sequence classification tasks, identification of motifs can potentially increase prediction accuracy (i.e., promoter identification [37]). Although gapped k-mer frequency features with different gap values can provide information regarding presence, absence, or density of various motifs in a sequence or sub-sequence, these frequencies fail to provide positional information of these motifs. This can provide classification models with additional discriminatory information since these motifs often demonstrate positional preferences [38]. To address these limitations, instead of using solely frequency-based k-mer and gapped k-mer features, in this study, we propose a novel feature extraction approach called “*Position-Specific Gapped k-mer*” with three distinct modes of feature generation. The following three subsections explain these modes. The names for different modes and equations used for providing a generalized description for the patterns within those modes heavily rely on several symbols, which are described in Table 1.

**Table 1:** A brief explanation of Symbols used in equations and Feature Mode names.

Symbols	Description
[AN]	A don’t care nucleotide (can be any one of the four nucleotides) within a pattern. These don’t care nucleotides are the **gaps** that we have briefly discussed so far.
FN	A nucleotide that is fixed (not a don’t care) within a pattern.
G	The number of gaps (don’t care nucleotides) within a pattern.
n	The number of possible patterns within a specific generation mode.
$f_{i}^{j}$	The binary indicator resulting from matching the j^th^ consecutive-nucleotide-pattern within a specific mode with the i^th^ consecutive-residue-group of length *M* along the RNA sequence. Here, M = length of the n^th^ pattern.
${\mathrm{RG}}_{i}$	The i^th^ consecutive-residue-group of length M along the RNA sequence.
M	A mono-mer. This is a k-mer with k = 1. This 1-mer is a pattern by itself in case of “Position-Specific k-mer” (no gaps). In case of “Position-Specific Gapped k-mer”, this 1-mer constitutes a larger consecutive-nucleotide-pattern of length M (M>= 2*k).
D	A di-mer. This is a k-mer with k = 2. This 2-mer is a pattern by itself in case of “Position-Specific k-mer” (no gaps). In case of “Position-Specific Gapped k-mer”, this 2-mer constitutes a larger consecutive-nucleotide-pattern of length M (M >= 2*k).
T	A tri-mer. This is a k-mer with k = 3. This 3-mer is a pattern by itself in case of “Position-Specific k-mer” (no gaps). We have not used this symbol in any mode of “Position-Specific Gapped k-mer”.

#### 3.1.1 Ps-Mono(G-gap)DiMer

The first feature generation mode in *Position-Specific Gapped k-mer* is called *Ps-Mono(G-gap)DiMer* or *PsM(G)D* in short. As the name *PsM(G)D* suggests, each of the patterns in this mode contains two k-mers (first one with k = 1 and the second one with k = 2) separated by G gaps. The following equation can express the patterns in this mode:

(2) $${\mathrm{PsM}(G)D}^{j}={\mathrm{FN}}_{1},\left(\left[AN\right]*\left\{G\right\}\right),{\mathrm{FN}}_{2},{\mathrm{FN}}_{3}$$

where $\mathrm{FN}\in \left\{A\left(adenine\right),C\left(cytosine\right),G\left(guanine\right),T\left(thymine\right)\right\}$, $\left[AN\right]=A\left|C\right|T|G$, $j\in \left\{1,2,\cdots ,n\right\}$, $G\in \left\{1,2,3\right\},$ & $n=4^{3}*3=64$.

Example of three patterns in this mode is, the mono-mer ‘A’ and the di-mer ‘CG’ separated by 1, 2 or 3 gaps which constitute the patterns ‘A-CG’, ‘A--CG’, and ‘A---CG’, respectively where the gaps ‘-‘ can be any of the four nucleotides. So, this mode contains 4^3^ = 64 length-4 patterns, 64 length-5 patterns and 64 length-6 patterns resulting from G = 1, G = 2 and G = 3, respectively. Each of these patterns can be matched with *L* – {(*G + F*) − *1*} consecutive-residue-groups along the RNA sequence where *L* is the length of RNA sequence, *G* is the number of gaps in the pattern, and *F* is the number of fixed nucleotides in the pattern. This process generates, (41-3) * 64 (where, G = 1) + (41-4) * 64 (where, G = 2) + (41-5) * 64 (where, G = 3) = 7104 binary indicators which can be defined using the following equation:

(3) $$f_{i}^{j}=\left\{\begin{array}{c} 1,if{\mathrm{RG}}_{i}={\mathrm{PsM}(G)D}^{j}\\ 0,otherwise \end{array}\right)$$

#### 3.1.2 Ps-DiMer(G-gap)Mono

Similarly, patterns in the second mode which is *Ps-Di(G-gap)MonoMer* or *PsD(G)M* in short, can be expressed through the following equation:

(4) $${\mathrm{PsD}(G)M}^{j}={\mathrm{FN}}_{1},{\mathrm{FN}}_{2},\left(\left[AN\right]*\left\{G\right\}\right),{\mathrm{FN}}_{3}$$

where $G\in \left\{1,2,3\right)\}$& $n=4^{3}*3=192$.

This mode contains 192 patterns (64 patterns with length 4, 64 length-5 patterns, and 64 length-6 patterns), which results in 7104 position-specific binary indicators. These indicators can be defined using an equation similar to Eq. (3).

#### 3.1.3 Ps-Mono(G-gap)Mono(G-gap)Mono

Lastly, patterns in the third mode which is *Ps-Mono(G-gap)Mono(G-gap)MonoMer* or in short *PsM(G)M(G)M,* is expressed through the following equation:

(5) $${\mathrm{PsM}(G)M(G)M}^{j}={\mathrm{FN}}_{1},\left(\left[AN\right]*\left\{G\right\}\right),{\mathrm{FN}}_{2},\left(\left[AN\right]*\left\{G\right\}\right),{\mathrm{FN}}_{3}$$

where G=1 & $n=4^{3}*1=64$.

This mode contains 64 patterns with length 5, which results in 2,304 position-specific binary indicators. These indicators can be defined using an equation similar to Eq. (3).

Hence, by applying the three modes of “Position-Specific Gapped k-mer” technique, we generated 7,107 + 7,107 + 2,304 = 16,512 binary features.

### 3.2 Position-Specific K-mer

The patterns considered while extracting “Position-Specific k-mer” [39] or *Ps-k-mer* features are traditional k-mers with different values of k (length of the patterns) with no gaps. In this study, we have considered k = 1, 2, 3 and the generated binary features have been categorized as *PsM*, *PsD* and *PsT* while the related patterns can be expressed using the following Eqs. (6), (7), (8), respectively:

(6) $${\mathrm{PsM}}^{j}={\mathrm{FN}}_{1}$$

where j∈{1,2,3,n} & $n=4^{1}=4$.

(7) $${\mathrm{PsD}}^{j}={\mathrm{FN}}_{1},{\mathrm{FN}}_{2}$$

where j∈{1,2,3,⋯,n} & $n=4^{2}=16$.

(8) $${\mathrm{PsT}}^{j}={\mathrm{FN}}_{1},{\mathrm{FN}}_{2},{\mathrm{FN}}_{3}$$

where j∈{1,2,3,⋯,n} & $n=4^{3}=64$.

For each of the patterns, binary indicators are generated by matching the pattern with all possible consecutive-residue-groups along an RNA sequence, which can be defined using an equation similar to Eq. (3). For example, if a pattern within *PsD* is ‘GG’, then for the sequence ‘ACGGCGGUG’, the vector representation will be ‘00100100′. If a pattern within *PsT* is “ACG”, then for the sequence ‘GUAGCACGG’ the vector representation is ‘0000010’. For each of the patterns, we get L – (k- 1) binary indicators where L = length of RNA sequence and k = length of the k-mer pattern. Therefore, for *PsM*, *PsD* and *PsT*, we get {41 - (1-1)}* 4 = 164, {41 - (2-1)}* 16 = 640 and {41 - (3-1)}* 64 = 2496 features, respectively.

### 3.3 GC – content

Guanine-Cytosine content, also known as GC-content, is one of the most powerful features for the identification of RNA modification sites [40], [41], [42], [43] due to its association with RNA stability [44]. For any given RNA sequence, GC-content is calculated as the percentage of the two nitrogenous bases, guanine and cytosine, in an RNA sequence. GC-Content of an RNA sequence can be expressed using the following equation:

(9) $$\frac{G+C}{A+C+G+U}*100$$

From the four modes described above and the GC-content, we generate a total of 19,877 features. The 19,876 binary features resulted from various patterns, and those patterns can be categorized into 10 types (3 types from *Ps-Mono(G-gap)DiMer,* 3 types from *Ps-Di(G-gap)MonoMer,* 3 types from *Ps-k-mer,* and 1 type from *Ps-Mono(G-gap)Mono(G-gap)MonoMer*. A summary of the number of features from each of these pattern types is provided in Table 2**.**

**Table 2:** Frequency distribution of features under different feature types and feature modes.

Feature Type	Feature Type Name	Number of Features	Feature Mode
FT1	PsM	164	*Position-Specific k-mer*
FT2	PsD	640	
FT3	PsT	2496	
FT4	PsM(1)D	2432	*Ps-Mono(G-gap)DiMer*
FT5	PsM(2)D	2368	
FT6	PsM(3)D	2304	
FT7	PsD(1)M	2432	*Ps-Di (G-gap)MonoMer*
FT8	PsD(2)M	2368	
FT9	PsD(3)M	2304	
FT10	PsM(1)M(1)M	2368	*Ps-Mono(G-gap)Mono(G-gap)MonoMer*
FT11	GC Content	1	*GC – content*

### 3.4 Optimized feature vector

In the case of any classical supervised model, as the number of features increases, while the number of training instances remains constant, the model’s generalization capability starts degrading after a certain point. This degradation occurs due to an increased number of control points of the model, while the number of constraints in the training data remains fixed, which enables it to capture idiosyncratic patterns from the training set. It, in turns, reduces the model’s interpolation capability. Moreover, if the features are highly correlated with one another, demerits of the high dimensionality outweigh the merits of the added information [45]. This often leads to a phenomenon called overfitting [46]. As our combined feature vector contained a comparatively large number of features (p ≪ n) after going through the extraction process described in the previous three sections, it became imperative to add a dimensionality reduction component in our framework for reducing the risk of overfitting.

**Fig. 2:**
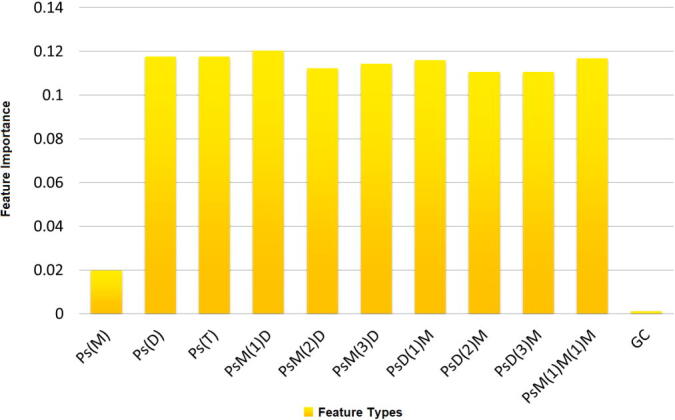
Feature importance of individual feature types.

For this purpose, we have performed feature selection using Random-Forest (RF) classifier, an ensemble model widely used for classification purposes [47], [48], [49], which can also return a ranking of the features based on their contribution in separating the instances belonging to different classes [50], [51], [52]. RF model is constituted of some unpruned decision trees (the number of trees is a hyperparameter of the model) trained in parallel with a two-level base-model-diversity ensuring mechanism, where each tree outputs a prediction for the instances, and the forest calculates the final prediction based on a majority-vote (voting over all the trees in the forest). We have used Gini impurity for selecting the splitters at the base-trees' internal nodes, and the average impurity decrease induced by the features across all the trees has been regarded as feature importance [53]. Fig. 2 shows the importance of the 11 feature types summarized in Table 2. It is to be mentioned that the 7 feature types generated from the 3 modes that are our novel proposals in this paper have been assigned equal or higher importance by the RF model compared to the 4 other types.

The conventional approach is to select a small subset of informative features directly from the original feature set. However, since 19,876 features (GC-content was preselected) in our combined set come from four generation modes, training the feature selector directly on that combined set returned a subset that resulted in suboptimal performance of the subsequent classification model in our initial experiments. This suboptimal performance can be attributed to the fact that if we train the selection model on a feature set of size *m* for identifying an informative subset of size *d* where, *d* ≪ *m*, the selected features are not equally distributed among the 4 feature generation modes. The probability of such outcome becomes even bigger since RF feature selector often assigns equal importance to correlated attributes, and the descriptors belonging to a specific generation mode can be correlated with one another while being equally informative individually. Although the members of such a skewed subset are individually informative, the subset can fail to provide the subsequent classification model with the diverse viewpoint required for maximizing class-separation, thus resulting in limited generalization capability.

For mitigating this issue, we have trained a separate RF model on each of the four feature modes, and 100 top ranking features returned by each of those selectors are combined for getting the reduced feature set of size 400. Additionally, GC-content is added to this set, which resulted in the final feature set with 401 features, that is used for training and evaluating the classification models. It is to be mentioned that, we have only used training sets for training the feature selectors in all cases for avoiding overestimation. In the case of k-fold cross-validation, feature selectors were trained inside the loop for ensuring that the validation sets are never used in the training process.

We have performed 10 times 5-fold cross-validation experiments (average of 10 cross-validation rounds) using a Logistic Regression (LR) model with no hyperparameter tuning (using default parameter to avoid over tuning) for assessing the contributions of the features from different modes which is reported in Table 3. We tested all possible combinations of the 4 modes (15 combinations in total) and observed that each of our 3 proposed modes (denoted by B, C, and D in the table) increased the classifier's performance. Note that, for each of the combinations, GC-content was added to the feature set while training the classification models and dimensionality reduction was performed using the abovementioned approach, which ensured mode-wise uniformity of the selected features.

**Table 3:** 50 times 5-fold cross-validation on each feature mode and their combinations, where A = Ps-k-mer, B = PsM(G)D, C = PsD(G)M, D = PsM(G)M(G)M).

Feature Mode	Accuracy	Precision	Sensitivity	Specificity	F1-Score	MCC
A	66.8	66.9	66.6	66.9	0.66	0.33
B	71.6	70.3	74.7	68.55	0.72	0.38
C	71.9	70.8	74.6	69.2	0.72	0.42
D	64.8	63.8	69.2	60.4	0.66	0.30
A + B	73.1	72.9	73.4	72.8	0.73	0.40
A + C	72.5	71.8	74.3	70.7	0.72.	0.44
A + D	70.7	70.5	71.4	70	0.70	0.40
B + C	76.6	77.7	77.5	77.6	0.77	0.51
B + D	74.5	73.8	76.2	72.8	0.74	0.47
C + D	73.6	72.9	75.1	72	0.73	0.41
A + B + C	76.9	76.7	77.5	76.4	0.77	0.50
A + B + D	74.9	74.8	75.5	74.4	0.75	0.47
A + C + D	74.7	74.4	75.5	73.7	0.74	0.45
B + C + D	77.2	77.5	79.7	76.6	0.78	0.54
A + B + C + D	**78.3**	**78.0**	**80.0**	**79.5**	**0.78**	**0.56**

In Table 4**,** the contributions of our proposed position-specific features have been analyzed using a downstream classification model’s performance. Additionally, we have performed 5 times 5-fold cross-validation experiment for identifying specific patterns from modes A, B, C, and D that contain significant discriminatory information. In this experiment, on each training partition (total 5*5 = 25 training partitions in 5 cross-validation rounds), 4 mode-wise feature selectors are trained on all modes (A + B + C + D) according to the procedure described above. This process returned 5 selected sets, each containing 400 position-specific features. Next, an intersection operation among these 25 sets returned 34 features that were present in every selected set. The 34 nucleotide patterns related to these 34 features and the position of the consecutive-residue-groups along RNA sequences that were matched with these patterns for generating the binary indicators are reported in Table 4.

**Table 4:** Analysis of frequent patterns and their position in RNA sequences.

Pattern	Start Position-End Position	Pattern	Start Position-End Position
**A-A-A**	22–26	**G-G-G**	36–40
**A-AA**	17–20	**G-G-U**	35–39
**AC---G**	28–33	**G-GU**	15–18
**AU--U**	19–23	**G-U-A**	33–37
**AUC**	25–27	**GC—C**	33–37
**AU**	15–16	**GG—G**	32–36
**C**-**C**-**C**	25–29	**GG-A**	11–14
**C-CC**	38–41	**GGG**	23–25
**C-CU**	21–24	**GGG**	31–33
**C-U-A**	23–27	**G**	24
**CA---A**	21–26	**U---CC**	3–8
**CA-C**	24–27	**U—AG**	33–37
**CA-C**	6–9	**U—UA**	19–23
**CC--C**	2–6	**U-GA**	1–4
**CG-G**	21–24	**UG---A**	24–29
**G-G-G**	14–18	**UGC**	34–36
**G-G-G**	31–35	**UUU**	25–27

Since these 34 binary indicators were present in each of the 25 feature sets selected by RF models trained on different instance partitions, we hypothesize that the presence or absence of the patterns in specific positions of an RNA sequence contains significant signal regarding 5-Hydroxymethylcytosine modification of the central residue. This hypothesis has been verified with an experiment on the independent data partition reported in Comparison with the State-of-the-art Methods Section. It is important to highlight that 27 out of these 34 features have been generated using different modes of our proposed position-specific gapped k-mer (modes B, C, and D), which retain significant discriminative information in the form of both short-range and long-range sequence-patterns.

## 4 Classification method

Since the main focus of this study is to propose new feature extraction mechanisms and establish their efficacy in identifying a specific type of RNA modification site, we have not focused on developing any problem-specific model architecture. We have employed LR [54], a widely used classifier on top of our optimized feature vector for learning the coefficients of a linear decision boundary in our derived feature space [55], [56], [57] that separates the 5hmC sites from the non-5hmC sites.

LR uses sigmoid function on top of an affine transformation step (the learnable parameters are related to this step) for estimating the *p* parameter of a binomial distribution that points towards the correct class for the instances. We have used LR implementation from the machine learning package scikit-learn [58]. It is to be noted that we have used the default hyperparameter settings provided by the package without any tuning from the validation set feedbacks inside k-fold cross-validation. Since the default optimizer is Limited-Memory Broyden–Fletcher–Goldfarb–Shanno (LBFGS) algorithm, there is no need to tune the learning rate (which would be essential in case of a gradient descent optimizer) since step-size is estimated from the Hessian matrix [59]. Additionally, since we already performed substantial dimensionality reduction as mentioned in the previous section, reducing overfitting through precisely tuning the regularization strength was not a requirement. Although tuning the penalty parameter could have resulted in a slightly better accuracy compared to the ones reported in the following section, we did not go for that since it could lead to some form of overestimation while calculating the k-fold cross-validation results for comparing with the current state-of-the-art method.

## 5 Performance evaluation

We used six different metrics, namely, Accuracy, Precision, Recall (Sensitivity), Specificity, F1-score, and Matthew’s correlation coefficient (MCC) to evaluate the prediction performance of our proposed framework. These metrics are defined as:

(10) $$\mathrm{Accuracy}=\frac{\mathrm{TP}+TN}{\mathrm{TP}+FP+FN+TN}*100$$

(11) $$\mathrm{Precison}=\frac{\mathrm{TP}}{\mathrm{TP}+FP}*100$$

(12) $$Recall\left(Sensitivity\right)=\frac{\mathrm{TP}}{\mathrm{TP}+FN}*100$$

(13) $$\mathrm{Specificity}=\frac{\mathrm{TP}}{\mathrm{TN}+FP}*100$$

(14) $$F1-score=\frac{2*Precision*Recall}{\mathrm{Precision}+Recall}$$

(15) $$\mathrm{MCC}=\frac{\left(TP*TN\right)-\left(FP*FN\right)}{\sqrt{\left(TP+FP)(TP+FN)(TN+FP)(TN+FN\right)}}$$

where True Positive (TP) represents the number of correctly identified 5hmC sites; True Negative (TN) represents the number of correctly identified non-5hmC sites; False Positive (FP) represents the number of non-5hmC sites incorrectly identified as 5hmC sites; False Negative (FN) represents the number of 5hmC sites incorrectly identified as non-5hmC sites.

We also used the area under the Receiver Operating Characteristic Curve (auROC) [60] and area under the Precision-Recall Curve (auPR) [61] to measure our predictor’s performance. For both of these metrics, the higher the area under the curve, the better the predictor is at distinguishing between 5hmC sites and non-5hmC sites. ROC curve plots TP against FP at different decision thresholds while the Precision-Recall curve plots the values of precision against the values of recall at thresholds.

We used 5-fold cross-validation [62] and an independent set to measure the generalization capability of our predictor. The process followed in this study to conduct 5-fold cross-validation is defined as follows:

1. The training dataset is randomly divided into five equal sub-datasets.
2. Four sub-datasets are used for training the predictor and the remaining sub-dataset is used for evaluating the predictor.
3. Repeat step 2 until each sub-dataset is used as test set once.
4. Repeat step 1 to step 3, 10 times before reporting the average results from those experiments.

Once the predictor is trained, we use it to perform prediction on the independent test set. The experimental results on cross-validation and independent test set are reported in the following Section.

## 6 Results and discussion

In this section, we first investigate the impact of LR compared to several other classifiers to build iRNA5hmC-PS. We then compare our results with those reported in previous studies and analyze the performance.

### 6.1 Comparison with different classification algorithms

Although our framework uses LR as the classification model, we experimented with two other models, namely, Support Vector Machine (SVM) with linear kernel [63] and Gaussian Naïve Bayes (GNB) [64] before finalizing on LR. We chose linear SVM because it finds a linear decision boundary on the original feature space like LR with some added constraints that direct the optimizer towards the boundary with maximum possible margin from the training instances [63]. GNB also served our purpose well since it is a simple generative model with the intrinsic property of not requiring any hyperparameter tuning, which we have maintained throughout all the experiments reported in this study. Results obtained on 5-fold cross-validation and the independent set for the three classifiers are reported in Table 5 and Table 6, respectively.

**Table 5:** Performance evaluation of LR, SVM, and GNB using 50 times 5-fold cross-validation.

Classifiers	Accuracy (STD)	Precision (STD)	Sensitivity (STD)	Specificity (STD)	F1-score (STD)	MCC (STD)
SVM	75.5 (0.41)	75.1 (0.50)	76.8 (0.09)	74.3 (0.79)	0.75 (0.30)	0.49 (0.29)
GNB	76.7 (0.34)	**83.3** (0.42)	69.6 (0.67)	**85.6** (0.35)	0.75 (0.47)	0.50 (0.40)
LR	**78.3** (0.48)	78.0 (0.19)	**80.0** (1.01)	79.5 (0.13)	**0.78** (0.59)	**0.56** (0.44)

**Table 6:** Performance evaluation of LR, SVM, and GNB using independent set.

Classifiers	Accuracy	Precision	Sensitivity	Specificity	F1-score	MCC
SVM	77.5	71.2	**78.8**	68.2	0.75	0.47
GNB	77	**76.2**	72.7	77.3	0.74	0.50
LR	**78.5**	76.1	77.3	**75.7**	**0.77**	**0.53**

From Table 5, we can see that LR achieves 78.3%, 78.0%, 80.0%, 79.5%, 0.78, 0.56 in terms of Accuracy, Precision, Sensitivity, Specificity, F1-Score, and MCC, respectively, outperforming the other two classifiers in four out of six metrics on 5-fold cross-validation. It is to be mentioned that, during our initial experiments, we tested the performance of different classification models available in scikit-learn library [58]. Among those classifiers, linear SVM, GNB, and LR demonstrated better predictive capability. Therefore, we have reported the results for those three models in this study. Superior performance of the simple linear models, specifically LR's performance (a simple linear classifier), demonstrates the class-discriminative information retention capability of our proposed features, which enables LR to effectively separate the 5hmC sites from non-5hmC sites using a linear decision boundary. In Table 6, we can see that LR outperforms SVM and GNB in four of the six metrics on the independent set, which is consistent with those results reported in Table 5. Consistency between the results reported in Table 5, Table 6 corroborate the generality of our model.

**Fig. 3:**
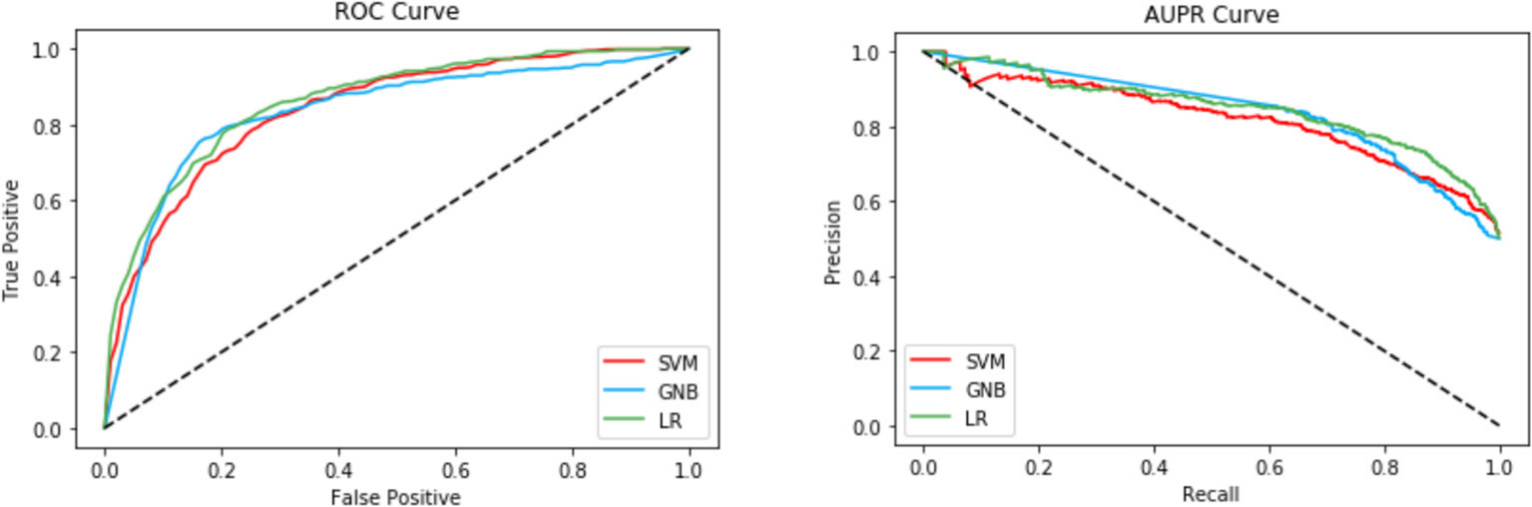
Performance evaluation of LR, SVM, and GNB using 5-fold cross-validation.

**Fig. 4:**
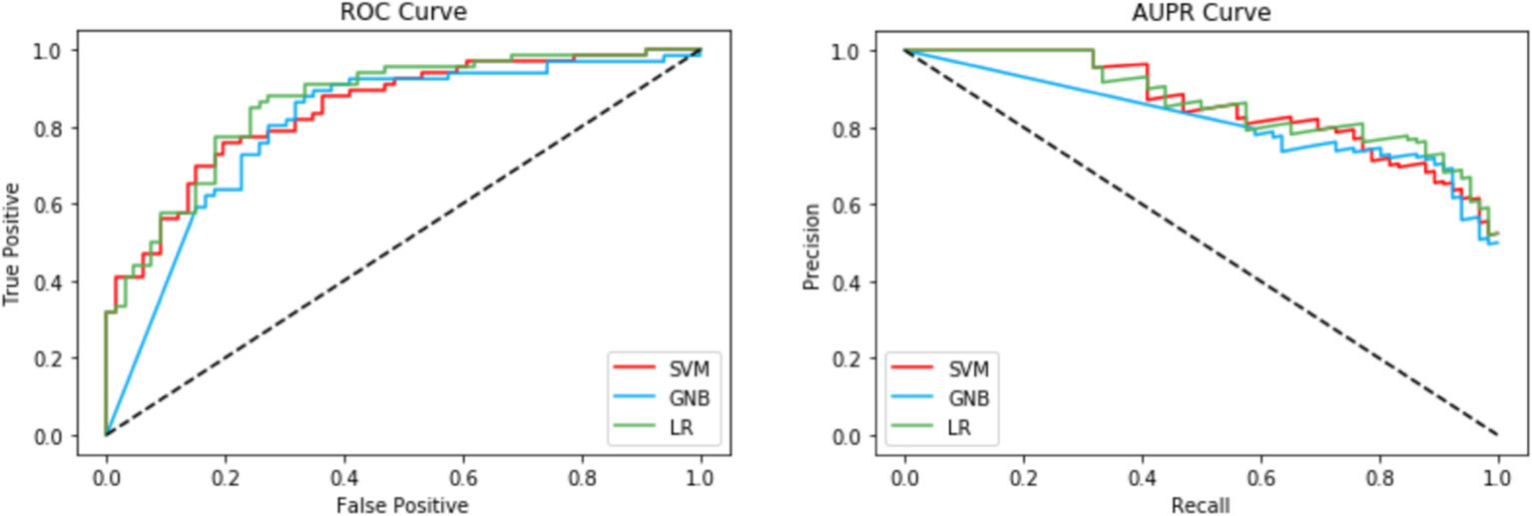
Performance evaluation of LR, SVM, and GNB using the independent test set.

We have also compared the performances of LR, SVM, and GNB using ROC and PR curves on both cross-validation and the independent set shown in Fig. 3, Fig. 4, respectively. In terms of auROC, LR, SVM, GNB achieves 0.86, 0.84, and 0.83 for cross-validation and 0.86, 0.84, and 0.80 on independent set. On the other hand, in terms of auPR, LR, SVM, GNB achieves 0.86, 0.83, and 0.81 in cross-validation and 0.87, 0.86, and 0.76 on the independent set. On both 5-fold cross-validation and independent test set, LR outperforms SVM and GNB, showing that LR has better discriminative power to distinguish the RNA 5hmc sites from the non-5hmC sites compared to the other two classifiers.

Since GC-content has not been subject to feature elimination (we have added it to our final feature set directly based on initial feature importance analysis reported in Fig. 2), we have performed additional 5-fold cross-validation experiments for SVM, GNB, and LR without taking GC-content into account to study the contribution of GC-content feature. The corresponding results are reported in Table 7. Comparing the results reported in Table 5 with those reported in Table 5 demonstrates the improvement in prediction performance by adding GC-content.”

**Table 7:** Performance evaluation of LR, SVM, and GNB using 5-fold cross-validation (our proposed feature set without GC-content feature).

Classifiers	Accuracy	Precision	Sensitivity	Specificity	F1-score	MCC
SVM	70.3	70.9	71.7	70.9	72.3	43.3
GNB	**76.7**	**82.1**	**76.3**	**85.1**	74.4	45.3
LR	76.3	74.6	74.2	82.3	74.2	**47.7**

Existing state-of-the-art method iRNA5hmC uses 1-mer, 2-mer, 3-mer frequency features along with position-specific 1-mer based binary features (these binary features are already included in our feature set). We have combined 1-mer, 2-mer, and 3-mer frequency-based features (4 + 16 + 64 = 84 k-mer frequency features) with our proposed and selected features (401 features) and performed cross-validation experiments using the combined feature set (485 features). The corresponding results are reported in Table 8. A comparison of these results with the ones in Table 5 shows that addition of k-mer frequency-based features degrades our feature set's performance for RNA 5hmC site prediction task. Thus, we have not included these frequency-based features in our current single-model based prediction pipeline.

**Table 8:** Performance evaluation of LR, SVM, and GNB using 5-fold cross-validation (our proposed feature set + features used in *iRNA5hmC*).

Classifiers	Accuracy	Precision	Sensitivity	Specificity	F1-score	MCC
SVM	72.9	72.2	**74.5**	71.3	73.3	42.2
GNB	**75.2**	80.7	66.6	**83.9**	**72.7**	**47.7**
LR	74.0	**74.1**	73.5	74.3	73.7	45.4

To study the significance of our proposed position-specific gapped k-mer features, we have performed cross-validation experiments using state-of-the-art gapped k-mer frequency-based features proposed in [33], and the corresponding results are reported in Table 9. Comparing these results with the ones reported in Table 5, suggests that our proposed position-specific features contain additional discriminative information required for detecting RNA 5hmC modification sites that previously proposed frequency-based counterparts did not to encapsulate. A comparison between results in Table 8 (our proposed position-specific gapped k-mer binary features + k-mer frequency features) and Table 9 (state-of-the-art gapped k-mer frequency features + k-mer frequency features) further suggests that the performance improvement observed in our framework iRNA5hmC is largely attributed to our proposed position-specific gapped k-mer based binary features and our proposed features cannot be substituted by existing state-of-the-art gapped k-mer frequency based counterparts without performance degradation.

**Table 9:** Performance evaluation of LR, SVM, and GNB using 5-fold cross-validation (k-mer + gapped k-mer frequency-based features only).

Classifiers	Accuracy	Precision	Sensitivity	Specificity	F1-score	MCC
SVM	62.2	61.5	64.6	59.7	62.6	22.2
GNB	59.9	58.7	66.1	53.7	61.2	16.1
LR	59.7	59.2	60.9	58.4	59.8	18.0

### 6.2 Comparison with the State-of-the-art method

**Fig. 5:**
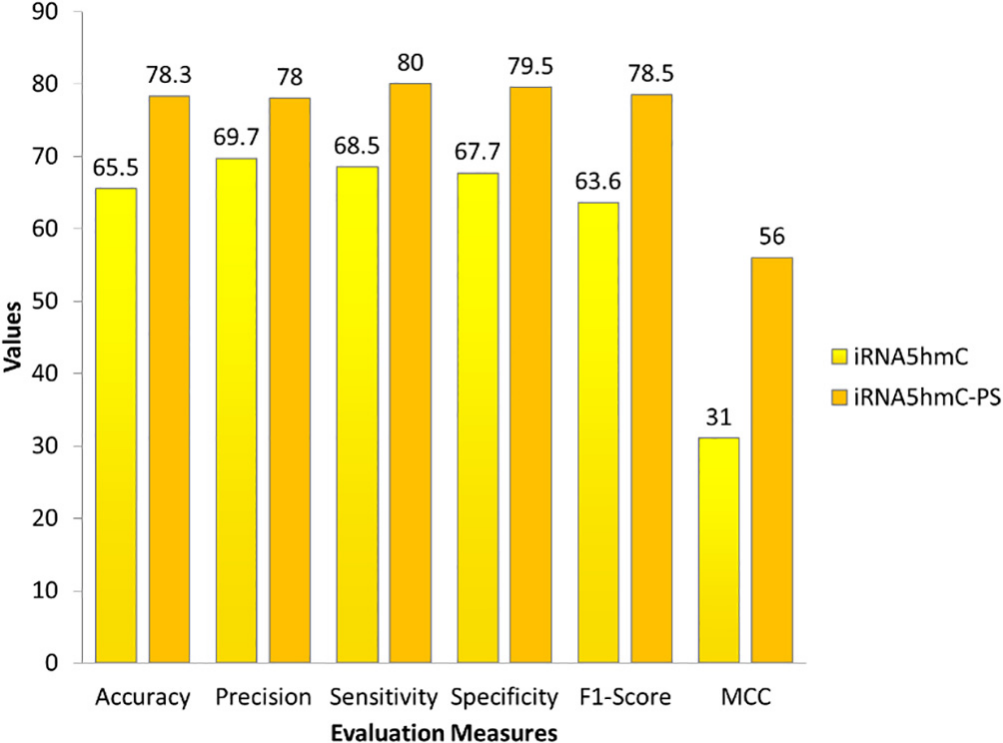
Performance comparison of iRNA5hmC-PS with iRNA5hmC achieved using 5-fold cross-validation. Note that we multiply auROC, auPR, and MCC by 100 to represent them on the same scale as the other evaluation measurements and the value of these measures have been provided along the y-axis (0–100).

In this section, we compare *iRNA5hmC-PS* with the existing state-of-the-art method, which is *iRNA5hmC*. *iRNA5hmC* uses different sequential information, namely, k-mer spectrum and nucleotide binary encoding. We evaluated *iRNA5hmC* and *iRNA5hmC-PS* on the same dataset using 5-fold cross-validation. The experimental result shown in Fig. 5 demonstrates that *iRNA5hmC-PS* significantly outperforms *iRNA5hmC. iRNA5hmC-PS* achieves 78.3%, 0.86, 0.86, 80%, 79.5%, and 0.56 in terms of Accuracy, auROC, auPR, Sensitivity, Specificity, and MCC, respectively. These results are 12.8%, 8.3%, 11.5%, 11.8%, 14.4%, and 25.0% higher compared to *iRNA5hmC* in terms of Accuracy, Sensitivity, Specificity, and MCC, respectively. These results demonstrate the superiority of *iRNA5hmC-PS* over *iRNA5hmC*.

Such promising performance with a substantially reduced feature set corroborates our hypothesis that the 34 frequently occurring position-specific indicators that are proposed in this study contain substantial discriminative information. Note that, as GC content is removed, all of the features used while acquiring the results in Table 7 mainly focuses on positional specificity between 5hmC and non-5hmC sites. Although these results are not as impressive as those in Table 5, they still substantially outperform *iRNA5hmC*. Therefore, features capturing positional specificity are much more significant than compositional features for separating RNA 5hmC from non-5hmC sites, which we believe is the main reason behind the superior performance of iRNA5hmC-PS over iRNA5hmC. Moreover, this finding establishes high interpretability of our framework, which can be useful in determining the biological functionalities of 5-Hydroxymethylcytosine modification. As a result, we believe iRNA5hmC-PS has the potential to be an accurate and efficient tool to identify 5hmC sites. iRNA5hmC-PS is publicly available as a web-server at http://103.109.52.8:81/iRNA5hmC-PS and benchmark dataset, source codes, and documentation for all the models are available at https://github.com/zahid6454/iRNA5hmC-PS. Detailed instructions on accessing and using different functionalities of the web-server is also provided in a readme file, online.

## 7 Conclusion

In this study, we propose a machine learning-based computational framework called iRNA5hmC-PS to identify RNA 5hmC sites. With our framework consisting of several novel sequence representation modes, a mode-wise feature selector and a predictor, we have significantly outperformed the current state-of-the-art method (iRNA5hmC), which had been established recently. For a fair comparison with *iRNA5hmC*, all our features are also extracted using primary sequence information. Our feature vector solely consists of position-specific binary indicators and the GC-content, whereas *iRNA5hmC* uses a combination of k-mer frequencies and position-specific information.

In fact, this study introduces a new paradigm with a promising predictive performance on a challenging problem by using RNA sequence representation consisting of position-specific binary indicators only. Although [18] demonstrated through feature analysis that RNA 5hmC sites are different from non-5hmC sites from both compositional and positional viewpoints, our empirical analysis has revealed that positional specificity between 5hmC and non-5hmC sites is much more significant compared to compositional specificity. Therefore, features capable of appropriately encoding this positional specificity is crucial for the appropriate classification of these sites. We believe these insights revealed through our study will be beneficial for developing more accurate computational frameworks for this problem. In our future work, we will explore the efficacy of our framework on other RNA modification site identification problems. Although, according to the experimental results, our proposed position-specific features do not work well in conjunction with existing frequency-based counterparts for RNA 5hmC site identification using single models, in the future, we aim to combine both frequency and position-specific features using our feature subspace ensemble approach proposed in [65] for different biological sequence-related problems. Moreover, unlike existing gapped k-mer frequency-based features and several other widely used features such as PseKNC [66], our proposed features do not obscure locality and relative ordering information of the residues while converting sequences to vectors. Therefore, our features are suitable for deep learning architectures consisting of convolutional and recurrent layers that exploit relative ordering of the residues for extracting discriminative patterns. In addition, we have deployed a useful webserver to make our framework easily accessible to the community. The webserver is publicly available at http://103.109.52.8:81/iRNA5hmC-PS and benchmark dataset, source codes, and documentation for all the models are available at https://github.com/zahid6454/iRNA5hmC-PS. Detailed instructions on accessing and using different functionalities of the web-server can be found in the readme file which is also available online.

## Author contributions

S. Ahmed, Z. Hossain, M. Uddin designed and performed the experiments. Z. Hossain developed the web-server. S. Ahmed, Z. Hossain, G. Taherzadeh, S. Shatabda, A. Sharma, A. Dehzangi wrote the manuscript. S. Ahmad, Z. Hossain, M. Uddin, A. Sharma, A. Dehzangi, G. Taherzadeh helped with figures and literature review. A. Sharma, G. Taherzadeh, A. Dehzangi, S. Shatabda mentored and analytically reviewed the paper. All the authors reviewed the article.

## Declaration of Competing Interest

The authors declare that they have no known competing financial interests or personal relationships that could have appeared to influence the work reported in this paper.
